# Sensitive, non-immunogenic *in vivo* imaging of cancer metastases and immunotherapy response

**DOI:** 10.15698/cst2023.08.288

**Published:** 2023-08-14

**Authors:** Joseph R. Merrill, Alessandra Inguscio, Taemoon Chung, Breanna Demestichas, Libia A. Garcia, Jill Habel, David Y. Lewis, Tobias Janowitz, Scott K. Lyons

**Affiliations:** 1 Cold Spring Harbor Laboratory, 1 Bungtown Road, Cold Spring Harbor, NY 11724.; 2 Cancer Research UK Beatson Institute, Garscube Estate, Switchback Road, Bearsden, Glasgow G61 1BD, UK.

**Keywords:** immunotherapy, cancer, imaging, SPECT, luciferase, sodium iodine symporter, metastases, reporter transgene

## Abstract

Non-invasive imaging of tumors expressing reporter transgenes is a popular preclinical method for studying tumor development and response to therapy *in vivo* due to its ability to distinguish signal from tumors over background noise. However, the utilized transgenes, such as firefly luciferase, are immunogenic and, therefore, impact results when expressed in immune-competent hosts. This represents an important limitation, given that cancer immunology and immunotherapy are currently among the most impactful areas of research and therapeutic development. Here we present a non-immunogenic preclinical tumor imaging approach. Based on the expression of murine sodium iodide symporter (mNIS), it facilitates sensitive, non-invasive detection of syngeneic tumor cells in immune-competent tumor models without additional immunogenicity arising from exogenous transgenic protein or selection marker expression. NIS-expressing tumor cells internalize the gamma-emitting [^99m^Tc]pertechnetate ion and so can be detected by SPECT (single photon emission computed tomography). Using a mouse model of pancreatic ductal adenocarcinoma hepatic metastases in immune-competent C57BL/6 mice, we demonstrate that the technique enables the detection of very early metastatic lesions and longitudinal assessment of immunotherapy responses using precise and quantifiable whole-body SPECT/CT imaging.

## INTRODUCTION

The immune system plays an integral role in both the development and treatment of metastatic cancer [1]. Although new cancer immunotherapy treatments have proven highly effective as systemic therapy for a subset of locally advanced and metastatic tumor types when previously only limited treatment options were available [2, 3], it has not proven universally effective. Immune-evasive mechanisms related to cancer cells [4, 5], the tumor microenvironment [6], systemic biological immune suppression [7, 8], and iatrogenic immune suppression [9, 10], contribute to the fact that the majority of patients with cancer do not yet benefit from cancer immunotherapy.

Accurate preclinical tumor models and non-invasive imaging techniques enable discovery of the molecular basis of the metastatic process and provide translatable insights into immunotherapy treatments. The best such cancer models recapitulate both genetic and phenotypic features of the human disease [11, 12]. On a practical level however, early detection of small lesions that recapitulate clinical scenarios and unpredictable body locations of systemic metastatic tumor development can make such models challenging to work with. Imaging enables repeated measures of tumor size and disease burden within the same individual over time and is therefore used in preclinical and clinical settings to assess treatment response [13]. However, not every whole-body imaging approach can confer sufficient sensitivity, resolution and precision to reliably detect and resolve individual small, non-superficial metastatic tumors spread throughout the body [14].

Reporter transgenes can substantially enhance image sensitivity, but commonly-employed reporters (e.g. GFP or firefly luciferase) originate from species other than the mouse. Such transgenes have been shown to be immunogenic in the context of immune-competent recipient mice, profoundly influencing *in vivo* tumor biology [15, 16]. Others have addressed this problem with the development of new transgenic mouse strains that constitutively express the same imaging reporters at spatially-distinct body locations. In most instances, signal from the inherited reporter alleles minimally interfere with tumor imaging and crucially, these mice develop central immune tolerance to the reporter transgene. Given the prevalence of reporter transgenes in preclinical research, these are important studies, but on a practical level, the maintenance of a transgenic colony can be both time-consuming and inflexible. Cohorts of tumor recipient mice need to be bred, genotyped and possibly back-crossed to optimally match the genetic background of the host with the engrafted tumor cells.

Here, we describe the development of a new tumor cell labeling vector called “immunostealth” that permits highly sensitive and tomographic imaging of metastatic tumor development *in vivo* without immunogenic consequences or the need to establish a transgenic breeding colony. This non-germline approach first relies upon the stable introduction of constitutive and tumor specific expression of murine NIS (mNIS; sodium iodide symporter; Slc5a5), which can confer sensitive *in vivo* tumor imaging via the uptake of radioactive isotopes of iodide or their radioanalogues [17]. Use of tomographic imaging techniques such as SPECT (single photon emission computed tomography) with [^99m^Tc]sodium pertechnetate ([^99m^Tc]NaTcO_4_) [18] or PET (positron emission tomography) with [^18^F]tetrafluoroborate ([^18^F]TFB) [19] permits non-invasive, sensitive and 3D visualization of NIS-labeled cells within deep tissue. As an endogenous gene, the expression of mNIS protein should not be immunogenic in the context of an immune-competent host.

Selection markers that confer antibiotic resistance or fluorescence are also frequently included in cell labeling vectors to enable efficient selection of stable reporter transgene expressing cells *in vitro* (e.g. puromycin *N*-acetylatransferase (PAC) [20] or GFP for flow-sorting or microscopy [21]). As these selection marker proteins originate from species other than the mouse, their expression *in vivo* will potentially be immunogenic, negating the benefit of mNIS utilization. To overcome this issue, we flanked the selection markers in the vector with FRT (flippase recognition target) sites [22], such that the markers can be efficiently and permanently removed by Flpo recombinase expression after *in vitro* selection and prior to *in vivo* experimentation.

Transient Flpo expression can be readily achieved *in vitro* by transduction with a commercially available adenoviral vector. Removal of the selection cassette from stably transduced cells leaves only mNIS transgene expression, meaning that labeled cells can be implanted and resultant tumors imaged sensitively in an immune-competent syngeneic host without inadvertently and artificially enhancing tumor immunogenicity. We demonstrate construct functionality and the ability of the technique to detect and measure the development of individual pancreatic cancer metastases with dimensions as small as one millimeter *in vivo*. We also use it to evaluate the effects of immunotherapy. Taken together, we demonstrate superior metastatic tumor imaging performance of mNIS based SPECT/CT over popular optical approaches.

## RESULTS

### Construction of the reporter vector

**Figure 1:**
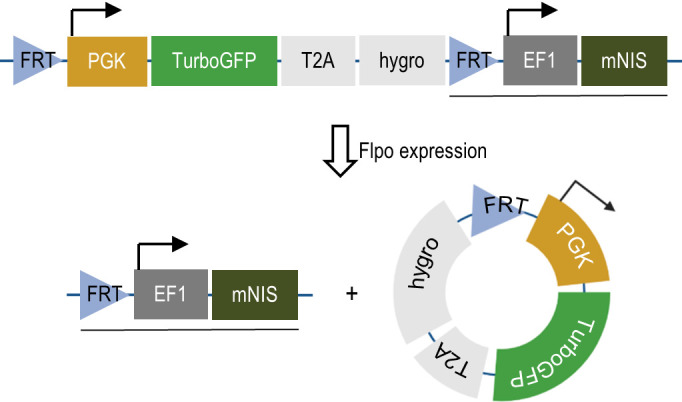
FIGURE 1: The immunostealth vector for mNIS-labeling of cells. A schematic illustration of the vector used to establish stable mNIS expression in tumor cells without potential immunogenicity arising from selection marker expression. Abbreviations; FRT – flippase recognition target. Flpo – flippase. PGK - phosphoglycerate kinase 1 promoter. TurboGFP – fast-maturing green fluorescent protein. T2A – self-cleaving 2A peptide. Hygro - hygromycin B phosphotransferase. EF1 – elongation factor-1 alpha promoter. mNIS – murine sodium iodide symporter (Slc5a5). Note; underlined transgenic sequence remains stably integrated in the transduced cell genome following Flpo recombination.

**Figure 2:**
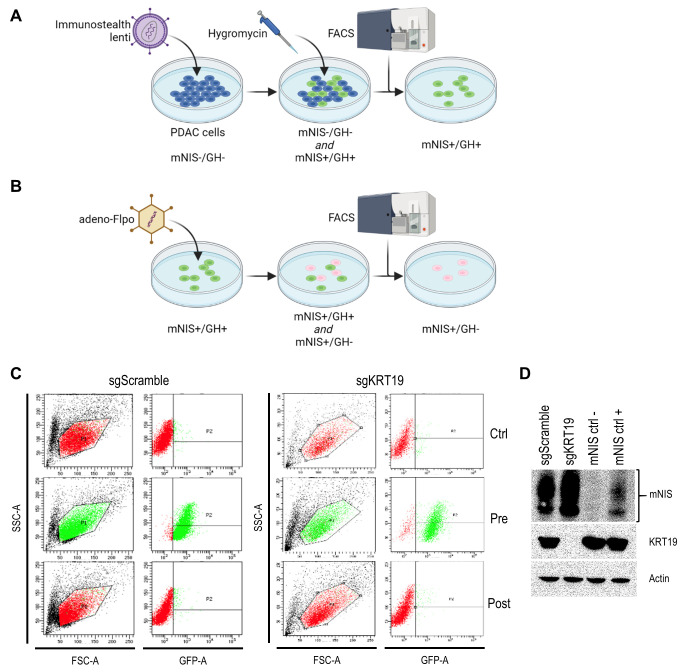
FIGURE 2: Transfection and selection of mNIS expressing KRT19 edited and control pancreatic cancer cells. (**A** and **B**) Schematic illustration of the workflow to obtain stable mNIS expression in transduced tumor cell lines without additional expression of potentially immunogenic *in vitro* selection markers. **(C)** Flow cytometry of expanded monoclonal sgKRT19 and sgScramble cancer cells prior to transduction (Ctrl), then before (Pre) and after (Post) adeno-Flpo transduction. **(D)** Western blot analysis for mNIS and KRT19 expression in monoclonal sgKRT19 and sgScramble cells following Ad-Flpo transfection and selection for the absence of GFP fluorescence (left two lanes). Blotting results on protein extracts from both non-mNIS expressing cells (NIS ctrl-) and mNIS expressing cells (NIS ctrl+) are shown in the right two lanes.

We developed the vector to sensitively and tomographically image syngeneic tumors developing in an immune-competent host without concern of reporter transgene expression eliciting an immune response and inadvertently influencing tumor biology. The vector comprises two expression cassettes (**Figure 1**). One cassette has a green fluorescent protein (TurboGFP) gene and the gene encoding hygromycin B phosphotransferase (which mediates hygromycin resistance in cell culture), expressed under control of the constitutively active phosphoglycerate kinase 1 (PGK) promoter. These transgenes allow efficient *in vitro* selection of stably transduced cells with antibiotics or by fluorescent cell sorting, but would be immunogenic in the context of tumor development *in vivo*. Accordingly, we flanked this selection cassette with FRT sites to enable its efficient removal with transient Flpo expression *in vitro* after initial selection (**Figure 2**). The second cassette constitutively expresses mNIS cDNA under control of the murine elongation factor-1 alpha (EF-1) promotor for non-invasive *in vivo* SPECT or PET imaging.

### Selection of transduced pancreatic cancer cells for stable mNIS expression

It was recently shown that tumors derived from murine pancreatic ductal adenocarcinoma (PDAC) cells respond differently to T cell targeted checkpoint immunotherapy with anti-programmed death receptor 1 (anti-PD1) blocking antibodies on the basis of keratin 19 expression [23] which impairs CXCL12 linkage and CXCR4 mediated T cell exclusion from tumors [24, 25]. To visualize this differential treatment response, we stably transduced KRT19 knockout and wild-type control PDAC cells (sgKRT19 and sgScramble respectively; kind gift from Douglas Fearon) with our lentiviral vector (**Figure 2**).

Stable full-length vector-expressing PDAC cells were first selected in hygromycin-containing media followed by single cell FACS (fluorescence-activated cell sorting) for GFP expression (**Figure 2A**). Next, mNIS positive, GFP/hygromycin positive monoclonal cells (mNIS+/GH+) were grown from single cell clones and transiently transfected with Flpo-recombinase expressing adenovirus (**Figure 2B**). This step efficiently removed the potentially immunogenic positive selection markers prior to *in vivo* experimentation. Following dilution cloning and the expansion of single clones, loss of GFP expression was confirmed by microscopy, FACS (**Figure 2C**) and PCR analysis. Finally, expression of mNIS in sgScramble and sgKRT19 cell lines was confirmed by western blot (mNIS+/GH-cells; **Figure 2D**).

### Whole-body non-invasive detection of individual metastatic lesions in deep tissue

**Figure 3:**
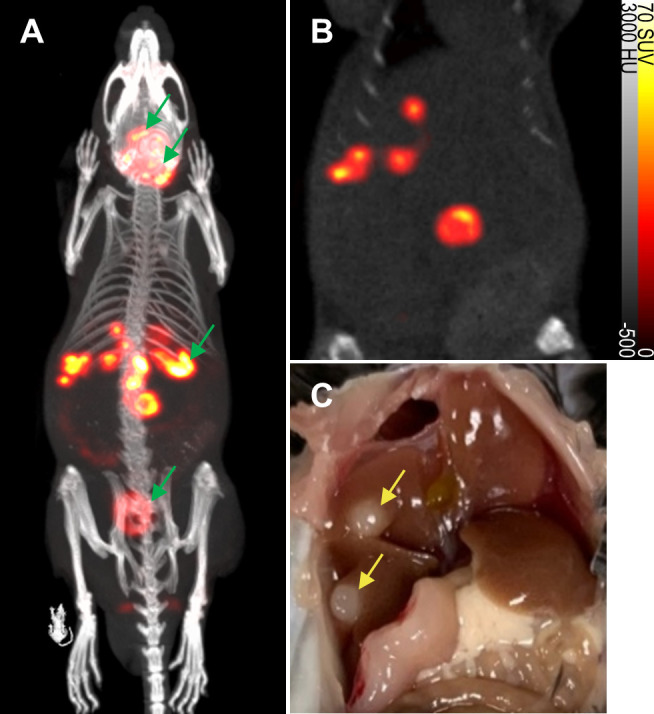
FIGURE 3: Detection of metastatic lesions *in vivo* by mNIS-SPECT. (**A** and **B**) A representative SPECT/CT maximum intensity projection (MIP) and a 2D coronal slice image of metastatic mNIS+/GH-pancreatic tumors developing predominantly in the liver, 5 weeks after tumor cell introduction via the portal vein. Note that the thyroid and salivary glands, stomach and bladder (denoted by green arrows in **A**) are sites of endogenous NIS expression or probe excretion and do not represent sites of tumor development. **(C)** Photograph of tumors (yellow arrows) in the liver of the same mouse imaged in A and B, taken 5 days later at necropsy (also see Figures S2 and S3).

To evaluate the non-invasive *in vivo* imaging performance of transduced mNIS-expressing cancer cells, mNIS+/GH-PDAC cells were injected via the portal vein into recipient syngeneic and immune-competent C57BL/6J mice. These mice subsequently develop fast-growing PDAC tumor metastases in the liver, reaching experiment end approximately 4-6 weeks after tumor cell injection (see **Figure 3** and Figures S2 and S3) [23].

To non-invasively monitor tumor progression, a SPECT scan was first taken 2 weeks after tumor cell introduction, then weekly to experiment end. The first time that metastatic lesions in the liver were evident by this method (subsequently used as enrollment criterion for treatment), we measured an average tumor-to-background liver ratio of 12.5 and average contrast-to-noise ratio of 82.6 (n = 6, data shown in **Table 1**) This level of image contrast far exceeds the Rose criterion, which states that the contrast-to-noise-ratio of an object or lesion must exceed 3 – 5 for it to be considered detectable [26] and demonstrates that our approach enables the assessment of small metastatic lesions at an early and experimentally useful time.

**Table 1.** Subject image quality parameters.

**Subject**	Tumor-to-background*	**Signal-to-noise#**	**Contrast-to-noise&**
NR-1	13.4	99.6	92.1
NR-2	8.12	62.8	55.1
NR-3	17.5	119.8	113.0
R-1	15.0	86.3	80.6
R-2	10.8	96.9	88.0
R-3	10.2	74.2	66.9
*Mean ± SD*	*12 5 ± 3 5*	*90 0 ± 20 1*	*82 6 ± 20 2*

* Tumor-to-background is the ratio of the mean intensity of the tumor ROI drawn as described divided by the mean intensity of a 4 mm diameter spherical ROI drawn in background normal liver: TBR = SUV_lesion_/SUV_background_

# Signal-to-noise is defined as the mean intensity of the tumor ROI divided by the standard deviation of the normal liver background ROI: SNR = SUV_lesion_/σ_background_

& Contrast-to-noise is defined as the mean intensity of the tumor ROI above that in the normal liver background ROI, divided by the standard deviation of the normal liver background ROI: CNR = (SUV_lesion_ − SUV_background_)/σ_background_ Averages are the arithmetic mean plus or minus the standard deviation.

Based on other SPECT experiments, we confirmed that mNIS-labeled lesions in the lung were submillimeter in size by anatomical CT when first detectable by SPECT. In this analysis, uptake volumes less than 1 mm^3^ (64 voxels) were filtered out of the region of interest to reduce false positive results (see materials and methods). Due to the partial volume effect, however, many of the metastatic tumors included in our analysis were likely between 0.1 mm^3^ and 1 mm^3^ in actual anatomic size.

### Longitudinal mNIS-SPECT imaging quantifies therapeutic response of metastases

**Figure 4:**
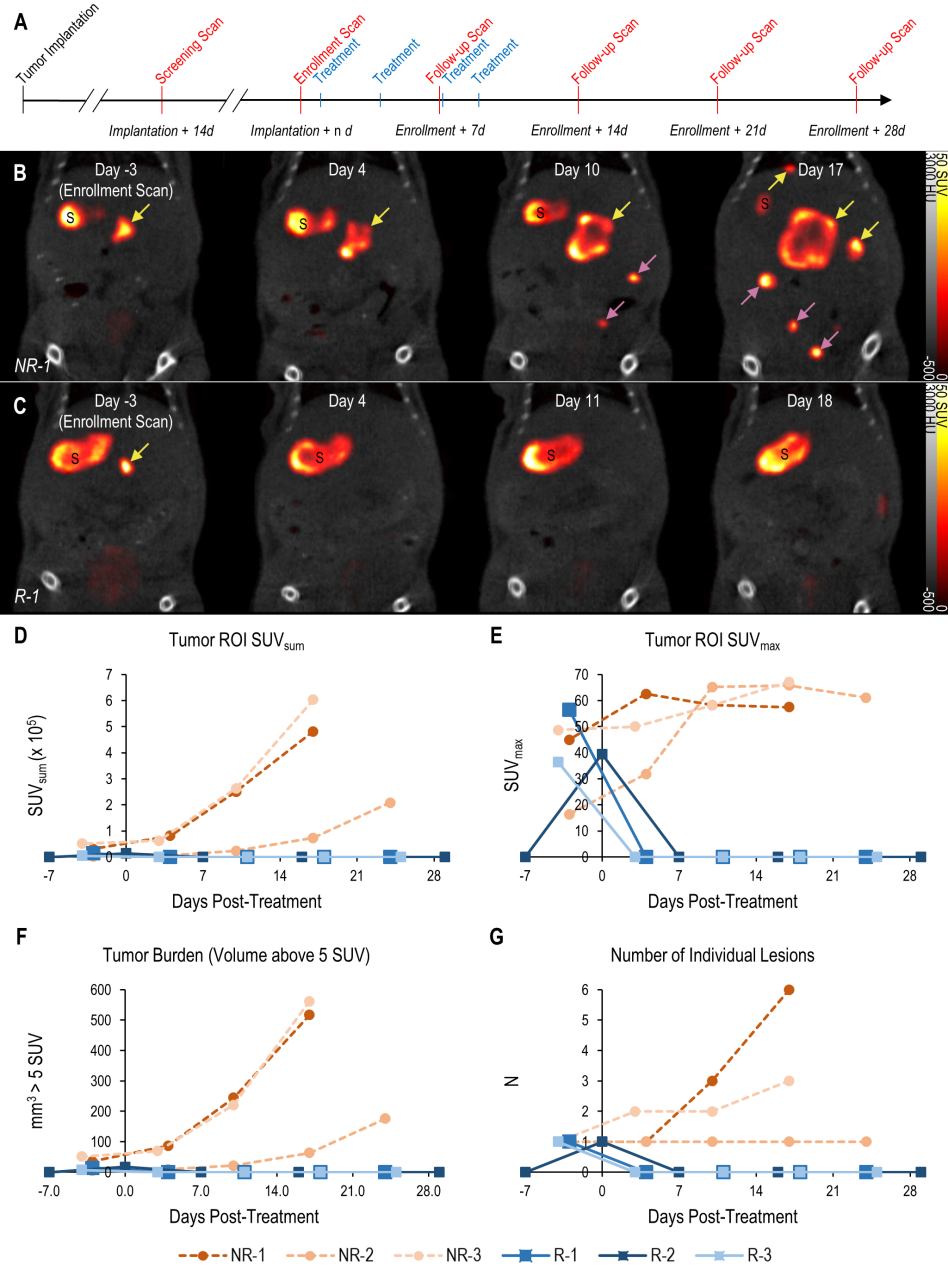
FIGURE 4: Treatment response monitoring of metastatic PDAC tumors using mNIS based SPECT/CT. (**A** and **B**) **(A)** Schematic experimental schedule for tumor implantation, imaging and drug treatment. **(B)** Coronal SPECT/CT images of representative mice from both experimental groups following implantation with wild-type or **(C)** KRT19 knockout tumor cells at: study enrollment (leftmost panels, day 15 post-implantation for NR-1 and day 14 for R-1); and following commencement of treatment (on day zero) with αPD-1. ‘S' indicates the stomach (an organ with high endogenous NIS expression). Yellow arrows indicate liver metastases, magenta arrows indicate metastases outside the liver (i.e. in the intestine). Response data for each subject following treatment determined using **(D)** SUV_sum_, **(E)** SUV_max_, **(F)** total apparent volume above 5 SUV threshold and **(G)** number of liver lesions. Subjects NR-1 – NR-2: sgScramble KRT19 wild-type tumor cells. Subjects R-1 – R-3: sgKRT19 knockout tumor cells.

The tomographic nature and high contrast-to-noise ratio of mNIS-SPECT imaging enabled us to take detailed images of individual metastatic lesion development and their response to αPD-1 immunotherapy (**Figure 4A**). Immediately after each scan, the images were quantitatively analyzed and mice with at least one 1 mm^3^ lesion in the liver (localized by co-registered CT) with a signal above 5 SUV were enrolled to start treatment with αPD-1 antibody. Tumor response to treatment was then followed with weekly scans for at least four weeks post-enrollment or until humane endpoint was reached for non-responders (**Figure 4**). The high contrast-to-noise of mNIS-SPECT imaging afforded an opportunity to analyze and present the treatment response data informatively in different ways. This included conventional SUV_sum_ and SUV_max_ measures (**Figure 4D and E**), as well as a proxy measure of tumor burden (quantifying the volume (mm^3^) of the ROI within the liver above the SUV 5 threshold (**Figure 4F**)), and the total number of individually resolvable lesions within the liver (as localized by co-registered CT (**Figure 4G**)). The combination of these latter two parameters categorize response to treatment using a scoring system analogous to the CT based Response Criteria for Solid Tumors (RECIST) [27] (see materials and methods), with response criteria based on apparent tumor volume and number of individual lesions as measured by mNIS-SPECT. Consistent with the literature, mice with sgKRT19 tumors (KRT19 knockout tumors, R-1, R-2 and R-3) showed radiological responses (partial or complete response) following αPD-1 treatment, whereas mice with sgScramble (KRT19 wild-type tumors; NR-1, NR-2 and NR-3) showed progressive disease (**Table 2**).

**Table 2.** Subject responses to αPD1 immunotherapy.

**Subject**	**Tumor Cell Line**	**Response 2 weeks post-Tx**	**Best Response**
NR-1	sgScramble	PD	PD
NR-2	sgScramble	PD	PD
NR-3	sgScramble	PD	PD
R-1	sgKRT19	CR	CR
R-2	sgKRT19	CR	CR
R-3	sgKRT19	CR	CR

PD: progressive disease; SD: stable disease; PR: partial response; CR: complete response

## DISCUSSION

Immunotherapies are now an established mainstay of cancer therapy and can cure metastatic cancers, such as melanoma, colorectal, lung, or bladder cancer [28–32]. However, not all tumor types are responsive and even within the more responsive cancer subtypes the majority of patients do not benefit from immunotherapy. Therefore, more preclinical and clinical research are needed [6, 33]. Non-invasive *in vivo* imaging can play a significant role in enhancing the quality of necessary preclinical research in murine model systems by enabling dynamic measurements of tumor development and response to experimental treatments. An important principle of molecular imaging, however, is that the imaging method employed should not fundamentally influence the biology being imaged. This principle is compromised in the context of imaging tumor development in an immune-competent host with the most commonly used reporter transgenes, such as firefly luciferase, that are immunogenic due to their origin from non-murine species. Indeed, several groups have clearly demonstrated that without immune-tolerization, the growth of tumors that express reporter transgenes such as firefly luciferase or GFP are significantly suppressed *in vivo* [15, 16].

The aim of this study was to develop a sensitive tumor imaging approach, capable of detecting small metastatic lesions spread throughout the body, without contributing artefactual tumor immunogenicity or requiring the maintenance of an immune-tolerized tumor recipient transgenic colony. Accordingly, we describe here the development of a tumor cell labeling lentiviral vector that delivers stable expression of a murine NIS transgene to transduced cells. As an endogenous protein, mNIS will not be antigenic in mouse tumor models. The vector also allows for efficient removal of markers after the selection of transduced cells, thus overall facilitating high sensitivity, high resolution tomographic imaging of labeled tumor cells by PET or SPECT without added immunogenicity.

The majority of reported preclinical studies that employ NIS as a reporter transgene have employed the human isoform, presumably with future clinical translation and gene therapy applications in mind. To prevent an immune response to NIS in immune-competent mice however, we have employed the murine NIS cDNA in our construct. Although not investigated directly by us, this may additionally confer some experimental advantages, as murine NIS protein has previously been shown to have higher activity and better membrane localization than the human isoform [34].

This metastatic tumor imaging approach offers a number of clear research advantages over other mainstream non-invasive approaches like CT (computed tomography), [^18^F]FDG-PET or BLI (bioluminescence imaging). (i) mNIS expression can be detected by SPECT (with [^99m^Tc]pertechnetate) [18] or PET (with [^18^F]TFB) [19] and as endogenous levels of NIS expression are extremely low or absent in most organs of the adult mouse (notable exceptions being the salivary glands, thyroid, lactating breast and stomach [35]), it offers very high image contrast of tumor lesions relative to normal tissue at most body locations. (ii) Unlike the visible photons detected by popular optical imaging techniques such as BLI, gamma radiation is minimally attenuated or scattered by overlying tissue. Unbiased tomographic whole-body scans are acquired, meaning that this imaging approach can detect labeled tumor cells at most, even unpredicted, body locations. (iii) NIS-mediated radiotracer uptake is indirectly ATP dependent [35] (a dependency similar to BLI with firefly luciferase), therefore necrotic or otherwise non-viable tumor cells will not contribute to signal. Thus, the apparent tumor volume as measured by SPECT is proportional to viable metastatic tumor burden, which is useful for therapeutic development. Unlike popular [^18^F]FDG-PET scans, NIS imaging signal is labeled tumor cell specific and is unaffected by inflammation or the local presence of metabolically active immune cells in the tumor microenvironment. Further, mNIS imaging is independent of the expression of possible targets of immunoPET or immunoSPECT approaches.

Taken together, mNIS-SPECT imaging is a sensitive and high-resolution approach, well-suited to imaging the spread of metastatic disease throughout the body. In our experience, mNIS-SPECT routinely enables detection of individual ≤1 mm^3^ sized lesions, largely irrespective of depth in tissue or proximity to other lesions. Response to treatment can be quantitatively measured from multiple independent cancer lesions within the same individual and no other whole-body imaging approach offers this combination of imaging sensitivity and resolution.

There are also limitations to this approach. PET/SPECT/CT imaging equipment is costly compared to BLI and an additional level of expertise is required to work with radiotracers. Scanning throughput is restricted to approximately 12 whole-body scans per day and SPECT, PET and CT imaging (routinely taken in parallel for attenuation correction and anatomic co-registration) all involve low, non-therapeutic doses of ionizing radiation that will accumulate over multiple scans. Particular to this study, the mNIS-labeled FC1242 cells also express Cas9 and so are unsuitable to demonstrate differences in tumorigenic potential between pre- and post-Flp'd mNIS-labeled cells. This is the most noticeable limitation of our study and will be addressed in future work.

The high signal-to-noise and high spatial resolution of NIS-SPECT imaging afforded us the ability to quantify the treatment response of individual metastatic lesions in a number of ways. Although SPECT is more closely related to PET, we based our categorization criteria off of clinical RECIST criteria and anatomical CT instead of the PERCIST criteria associated with FDG-PET. Although indirectly ATP-dependent, tracer uptake and signal intensity is primarily dependent on mNIS expression level and not determined by disease status or metabolic activity within the local tumor microenvironment. We therefore used an arbitrary cutoff for image intensity (5 SUV) to best eliminate false positive signal, then manually removed signal from normal organs that endogenously express NIS in the field of view (i.e. the stomach). The volume of the resultant ROI is not as accurate as one calculated from an anatomical imaging modality such as CT or MRI. However, the submillimeter spatial resolution of the small animal SPECT system permits a more accurate quantification than PET, and by comparing the total ROI volume in the same mouse over time we can control for errors in the apparent volume.

In conclusion, our imaging approach enables sensitive and tomographic *in vivo* imaging of individual metastatic lesions in an immune-competent host without contributing additional immunogenicity. The approach is readily transferrable to any murine cancer model system based on implanted cells and may aid future development of cancer immunotherapy strategies.

## MATERIALS AND METHODS

### Plasmid construction

The construct (**Figure 1**) was built into the pBOB lentiviral backbone (3^rd^ generation lentiviral vector) [36] to facilitate labeling and stable expression of murine NIS in tumor cell lines of interest. The *in vitro* selection cassettes (PGK promoter driven TurboGFP-T2A-puromycin resistance) flanked by FRT sites [22] were synthesized by GenScript USA (New Jersey). We used NCBI Reference Sequence: NP_444478.2 (Supplemental Figure S1) as the consensus coding sequence for murine NIS (Slc5a5). The FRT'd selection cassette and mNIS were cloned into the final construct in two sequential cloning steps. All restriction enzymes, T4 DNA ligase and taq DNA polymerase were supplied by NEB. All cloning and sequencing primers were supplied by Sigma (Burlington, MA). All plasmids were grown up in Top10 competent bacteria (Invitrogen, Waltham, MA) and plasmid DNA prepped from bacteria with Qiagen DNA mini prep or maxi prep kits (Germantown, MD).

### Production of lentivirus

Lentivirus was produced as per standard protocol [36] following the co-transfection of 293T cells with the immunostealth plasmid and three packaging plasmids (pMDL, pREV and pVSVG) in equimolar amounts. Lentiviral supernatant was collected 72 hours after plasmid co-transfection, filtered through a 0.45 µm filter and either used directly to transduce cells or frozen down at -80 °C in 1 ml aliquots.

### *In vitro* propagation of cancer cell lines and transduction with lentivirus

All cells were cultured in DMEM medium (Cellgro) supplemented with 10% FBS (Seradigm), 100 units/ml penicillin and 100 μg/ml streptomycin. Both cytokeratin 19 knockout (sgKRT19) and scrambled sgRNA control (sgScramble) variant subclones of FC 1242 cells (originally derived from KPC PDAC model mice [37] and kind gift from Douglas Fearon, CSHL [23]) were transduced with 500 μl immunostealth lentiviral supernatant. 72 hours later, stable integrants were selected in hygromycin containing media (300 μg/ml; #10687010, Invitrogen, Waltham, MA) for approximately 10 days. Resulting antibiotic resistant and single cell FACS sorted GFP positive cells were then further expanded in culture as stably transduced monoclonal populations and frozen down as aliquoted stock at -80 °C.

### Removal of *in vitro* positive selection markers

To remove potentially immunogenic selection markers, lentiviral transduced (mNIS+/GH+) sgScramble and sgKRT19 variant FC1242 cells were further transduced at 1000 MOI with Ad-CMV-Flpo (#1775, Vector Biolabs, Malvern, PA). 72 hours later, adenovirus transduced and GFP-negative cells were single cell FACS sorted and further expanded in culture. Fluorescence microscopy was first used to verify the loss of GFP expression. Removal of the positive selection cassette was further confirmed by PCR analysis, while the preservation of mNIS expression was assessed by Western blotting with an anti-NIS antibody (1:200 dilution; #514487, Santa Cruz, Dallas, TX).

### PCR

Reaction conditions to detect GFP DNA sequence, amplicon size 520 base pairs. Forward primer; GCCGCATGACCAACAAGA Reverse primer; TCGGTGTTGCTGTGATCC. PCR master mix prepared according Phusion High Fidelity DNA polymerase kit (#M0530, New England Biolabs Ipswich, MA). Final concentration of components in each reaction; 1X Phusion HF buffer, 200 μM dNTPs, 0.5 μM each primer, 200 ng template DNA, 1 unit of Phusion HF DNA polymerase and ddH_2_0 to a final volume of 50 μl. PCR cycled at 98°C for 30 sec, then 35 cycles at 98°C for 10 s, 62°C for 20 s, 72°C for 15s, then 5 mins at 72°C.

### *In vivo* model of metastatic PDAC to the liver

All animal protocols were approved by the Cold Spring Harbor Laboratory Institutional Animal Care and Use Committee. Hepatic PDAC tumor metastases were established in immune-competent male C57BL/6J mice (Jackson Laboratory, Bar Harbor, Maine; stock #000664) via portal vein injection [38] of 5 x 10^4^ mNIS-labeled sgScramble or sgKRT19 variant FC1242 cells.

### SPECT imaging of metastatic tumor burden

Metastatic tumors were imaged by mNIS-SPECT on a Mediso nanoScan SPECT/CT scanner (Mediso USA, Arlington, Virginia). Mice were injected intravenously via tail vein catheter with a nominal activity of 50 MBq [^99m^Tc]sodium pertechnetate, diluted in saline to a volume of 150 µl. Residue activity in the syringe and catheter was measured and subtracted from the total dose. After 50 minutes conscious uptake, mice were anesthetized with 3% isoflurane in oxygen, weighed and placed on a Mediso imaging cradle that monitored respiration rate and maintained body temperature with circulating warm air. Lubricating ophthalmic ointment (Dechra Puralube) was applied and 1 – 2% isoflurane anesthesia was maintained for the duration of the scan.

A CT scan (360 projections at 50 kVp and 192 µAs exposure) was first acquired for anatomical reference and attenuation correction. A SPECT scan with standard mouse pinhole collimators (Mediso APT62), encompassing a region from the lungs to the gut (transaxial FOV 33x33 mm, axial length of 26mm), was then acquired 60 minutes after [^99m^Tc]sodium pertechnetate injection (total SPECT scan time 10 minutes). CT images were reconstructed using filtered back projection with a cosine filter to a voxel size of 250 µm isotropic. SPECT images were reconstructed using a 3D iterative algorithm optimized for high dynamic range with 48 iterations and two subsets to a 128x128 matrix (258x258 µm pixel size, 258 µm slice thickness). Attenuation and scatter corrections were applied, radioisotope decay was corrected to image acquisition start time and raw counts were calibrated to activity concentration (Bq/ml).

### Image Analysis

Images were analyzed with VivoQuant 4.0 software (inviCRO, Boston, MA, USA) using a custom script pipeline (available upon request). An arbitrary threshold of 5 SUV was applied to highlight regions of increased uptake on the SPECT images. The stomach, an endogenous site of pertechnetate uptake, was manually removed from the thresholded region. Regions of uptake 64 voxels (1mm^3^) or greater localized within the liver by the coregistered CT image were included in the final region of interest (ROI), while smaller regions were filtered out to reduce false positives. Total apparent volume of the ROI (V_SUV5_), number of separate lesions (n), maximum uptake value (SUV_max_), and the summed uptake values of each voxel in the ROI (SUV_sum_) were quantified.

### Treatment of metastatic PDAC tumors

Upon enrollment on treatment, all mice received four doses of 200 μg rat anti-mouse PD-1 antibody (BioXcell, BP0273) diluted into PBS in a final volume of 200 μl per mouse, administered via intraperitoneal injection every 2 or 3 days.

### Treatment response criteria

The radiological response of each mouse following treatment was categorized utilizing a RECIST-like scoring system [27], with response criteria adapted for volume measurements and preclinical SPECT. Sustained disappearance on SPECT of all target lesions was considered complete response (CR). An increase of at least 73% in apparent volume of target lesions compared to the smallest volume at or after enrollment, or the appearance of any new lesions in the liver, were considered progressive disease (PD). A decrease of at least 66% in apparent volume, or a transient disappearance of lesions were considered a partial response (PR). Any response that did not fall into one of these categories was considered stable disease (SD). Longitudinal subject treatment response was tabulated for each post-treatment timepoint. A spherical ROI 4 mm in diameter was drawn in normal tissue in the median lobe of the liver on each enrollment scan for calculation of tumor-to-background, signal-to-noise and contrast-to-noise ratios.

## SUPPLEMENTAL MATERIAL

Click here for supplemental data file.

Click here for supplemental data file.

All supplemental data for this article are available online at www.cell-stress.com/researcharticles/2023a-merrill-cell-stress/.

## Abbreviations:

mNIS: – murine sodium iodide symporter,
SPECT: – single photon emission computed tomography,
FRT: – flippase recognition target,
GFP: – green fluorescent protein,
PGK: – phosphoglycerate kinase 1,
EF-1: – elongation factor-1,
PDAC: – pancreatic ductal adenocarcinoma,
anti-PD-1: – anti-programmed death receptor 1,
FACS: – fluorescence-activated cell sorting,
MIP: – maximum intensity projection,
RECIST: – Response Evaluation Criteria in Solid Tumors.
