# Supplementary material for: Sensitive, non-immunogenic in vivo imaging of cancer metastases and immunotherapy response
Source: Cell Stress. 2023 Aug 14;7(8):59–68. doi: 10.15698/cst2023.08.288 (PMC10468692; doi:10.15698/cst2023.08.288)
Supplement: Supplementary file 1 [file ces-07-059-s01.pdf]

Supplemental material for Merrill and Inguscio *et al*, “Sensitive, non-immunogenic in vivo imaging of cancer metastases and immunotherapy response.”

Supplemental Figure S1

**1**	MEGAEAGARATFGPWDYGVFATMLLVSTGIGLWVGLARGGQRSADDFFTGGRQLAAVPVG
**61**	LSLAASFMSAVQVLGVPAEAARYGLKFLWMCVGQLLNSLLTALLFLPIFYRLGLTSTYQY
**121**	LELRFSRAVRLCGTLQYLVATMLYTGIVIYAPALILNQVTGLDIWASLLSTGIICTLYTT
**181**	VGGMKAVVWTDVFQVVVMLVGFWVILARGVMLMGGPWNVLSLAQNHSRINLMDFDPDPRS
**241**	RYTFWTFVVGGSLVWLSMYGVNQAQVQRYVACHTERKAKLALLVNQLGLFLIVASAACCG
**301**	IVMFVYYKDCDPLLTGRIAAPDQYMPLLVLDIFEDLPGVPGLFLACAYSGTLSTASTSIN
**361**	AMAAVTVEDLIKPRMPSLAPRKLVFISKGLSFIYGSTCLTVAALSSLLGGGVLQGSFTVM
**421**	GVISGPLLGAFTLGMLLPACNTPGVLSGLTAGLAVSLWVAVGATLYPPGEQTMGVLPTSA
**481**	AGCTNASVLPSPPGAANTSRGIPSSGMDSGRPAFADTFYAVSYLYYGALGTLTTMLCGAL
**541**	ISYLTGPTKRSSLGPGLLWWDLARQTASVAPKEDTTTLEDSLVKGPEDIPAATKKPPGFR
**601**	PEAETHPLYLGHDAETNL

NCBI Reference Sequence NP_444478.2 for murine NIS (Slc5a5)

Supplemental Figure S2

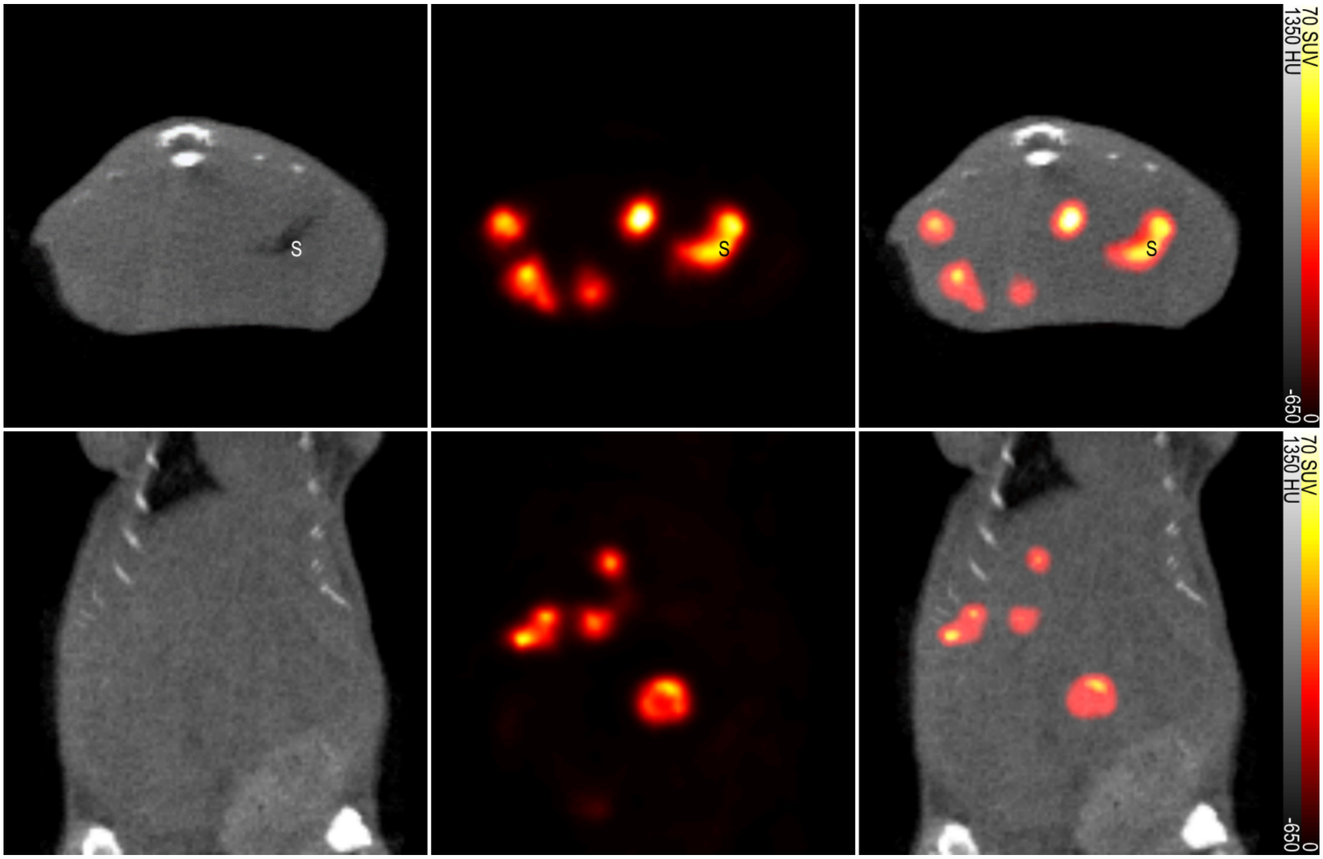

CT (left, grayscale), SPECT (middle, hot) and coregistered SPECT/CT (right) slices showing pertechnetate uptake in liver lesions developed following portal vein injection of immunostealth-labeled murine PDAC cells. Radiotracer is also taken up by NIS endogenously expressed in the stomach ('S'). CT window is set from -650 to 1350 HU. SPECT is set from 0 to 70 SUV.

Supplemental Movie S3

Maximum intensity projection (MIP) image of coregistered SPECT/CT images of the same subject as Figure S2. Endogenous NIS expression is seen in the stomach, salivary glands and thyroid, and radiotracer clearance in the urinary bladder. Hotspots in the thoracic region are mNIS-labeled individual metastatic lesions. MIP rendered from CT images with a window of -500 to 4000 HU and a SPECT window of 0 to 70 SUV.
