# Supplementary figures and images for: Sensitive, non-immunogenic in vivo imaging of cancer metastases and immunotherapy response
Source: Cell Stress. 2023 Aug 14;7(8):59–68. doi: 10.15698/cst2023.08.288 (PMC10468692; doi:10.15698/cst2023.08.288)

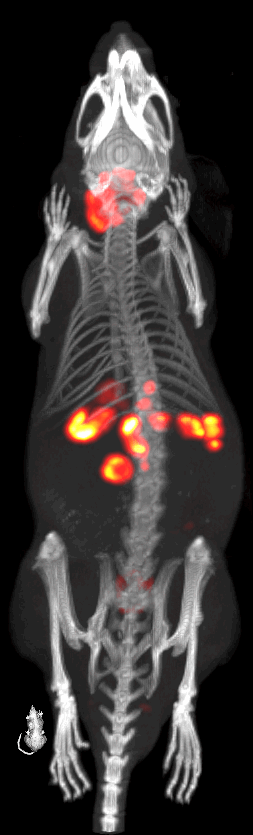

Supplement: Supplementary file 2 [file ces-07-059-s02.gif]
